# Foundational kelp species reveal links between host traits, the environment and the associated microbial community

**DOI:** 10.1098/rsos.250637

**Published:** 2025-10-15

**Authors:** Catherine A. Pfister, Emma Stanfield, Maximiliana Bogan, Brooke L. Weigel, Sativa Volbrecht, Kaylie Scorza

**Affiliations:** ^1^ Department of Ecology and Evolution, The University of Chicago, Chicago, IL, USA; ^2^ Committee on Evolutionary Biology, The University of Chicago, Chicago, IL, USA; ^3^ Oceans Department and Hopkins Marine Station, Stanford University, Palo Alto, CA, USA

**Keywords:** microbiome, carbon fixation, ocean warming, nutrient dynamics, host–microbe, dissolved organic carbon release, population structure

## Abstract

Canopy kelp are foundational species in coastal ecosystems and host diverse bacterial communities. Here, we test the association between bull kelp (*Nereocystis luetkeana*) host traits, blade-associated bacterial taxa and seawater environmental features across nine sites spanning more than 200 km in Washington state. Traits related to kelp fitness, environmental features and microbial community structure differed geographically. Kelp carbon fixation and tissue nitrogen content were greater at outer coast locales, compared with more inland locales in central and south Puget Sound. Geographic differences in carbon fixation rates, tissue nitrogen and bulb diameter were positively correlated with seawater nutrients and negatively correlated with sea surface temperature. Bacterial taxa showed differentiation among sites, and blade-associated bacterial densities were higher at the outer coast site compared with the most inland site. Yet, 11 bacterial genera were present in at least 80% of the samples; these taxa probably serve as core members of the *N. luetkeana* microbiome and show both positive and negative correlations with host health and environmental features. We show that there are strong interrelationships between kelp traits, seawater features and bacterial community composition with implications for the health of this highly productive foundational species in coastal ecosystems.

## 1. Introduction

Broadly distributed species can occur over strong environmental gradients and show differences in fitness along these gradients as a result [1,2]. In the context of global environmental change, these broadly distributed species can provide powerful natural systems for generating and testing hypotheses about the relationships between environmental variation and key aspects of organismal fitness. Local adaptation and plasticity are ways in which species respond to environmental variability and global change [3,4]. Foundational species in coastal marine systems can be distributed over thousands of kilometres and across areas with disparate sea surface temperatures and other environmental variables [1,5,6]. Close inspection of some foundational species reveals strong local differences in host fitness [1,7], as well as distinctions in host-associated microbes [8,9], strengthening the need to understand host–microbiome interactions across environmental gradients.

Many foundational primary producers have strong latitudinal patterns related to temperatures, including the global seagrass *Zostera marina* [10], oceanic cyanobacteria [11] and the members of the brown algae, known as kelp [12]. Ocean circulation and the features of coastlines can mimic latitudinal differences by producing a gradient in seawater temperatures, much in the same way that altitudinal gradients can mimic latitude in montane systems [13]. Changes in host fitness across environmental gradients can also alter the microbiome of the host [8]. In the study described here, distance from open, offshore areas to inshore fjord waters generates a gradient of increasing temperature, decreased nutrient supply and increasing severity of environmental change that greatly impacts the foundational bull kelp, *Nereocystis luetkeana*, and its diverse microbiome.

Environmental gradients can drive individual fitness consequences through numerous pathways for primary producers. In the foundational marine brown seaweeds known as kelp, direct effects of temperature can affect fitness-related traits including photosynthetic physiology [14], carbon fixation [15,16] and morphological development [17]. Decreased nutrients, often associated with warmer surface waters in the ocean, also reduce the photosynthetic capacity of kelp [16,18]. Decreased access to light, often as a result of turbidity from anthropogenically related run-off, decreases carbon fixation and overall metabolism of kelp [19]. Changes to kelp metabolism and physiology can have cascading ecological effects, including on kelp population size [20], interactions with grazers [21] and on local nutrient cycling [22,23]. Changes to the food web in coastal ecosystems can also alter kelp densities [24].

Environmental drivers of kelp fitness might also influence the observed differences in host-associated microbial communities across populations. Kelp have been shown to host taxonomically and functionally diverse microbes [8,25–29]. Microbes have metabolisms for using the unique complex carbon compounds produced by kelp [30,31]. *Nereocystis*-associated microbes metabolize dissolved organic nitrogen [32], including the most abundant amino acids in kelp [33], thus liberating inorganic nitrogen as ammonium in proximity to the host. Environmental stressors that alter kelp condition may generate feedbacks with host-associated microbes. Indeed, host condition had a dominant effect on the microbiome of an Australian kelp compared with surrounding environmental conditions over tens of kilometres of distance around Australia and Tasmania [8]. While kelp condition may influence the microbial community, the reciprocal is also possible if bacterial metabolisms aid the seaweed host. Warming temperatures can change bacterial composition [34], increasing the incidence of pathogenic bacteria and perhaps increasing host cell wall degradation by bacteria [35].

The canopy-forming bull kelp, *Nereocystis luetkeana*, in the northeast Pacific occurs from Alaska to California, USA. In the state of Washington, it forms extensive beds, or kelp forests, providing habitat for myriad species [24,36], producing up to 2.35 kg of biomass per square metre [37], and thus exerting strong biogeochemical effects on the surrounding seawater [38]. Indigenous people have relied on kelp for millennia [39]. *Nereocystis luetkeana* spans the outer coast region into the Strait of Juan de Fuca, north into the Salish Sea, and into the southernmost reaches of Puget Sound, a gradient of decreasing oceanic circulation and influence. Outer coastal and western areas of the Strait of Juan de Fuca experience seasonal upwelling events that bring cold nutrient-rich waters to the surface [40]. Eastward into the Strait of Juan de Fuca and Salish Sea regions, upwelling effects are dampened [41], resulting in lower dissolved inorganic nitrogen and salinity and higher temperatures [42–44]. Wave energy too is diminished eastward from the outer coast, further reducing the supply of nutrients to seaweeds [45]. Along the outer coastal regions and the western Strait of Juan de Fuca, bull kelp populations have been persistent, probably for over a century [46], even with alterations to the food web [24]. By contrast, populations in southern areas of Puget Sound have declined as much as 63% [47], a trend that may be related to many stressors, including increased seawater temperatures in Puget Sound [48]; kelp declines globally have been linked to warming seawater temperatures [49]. The bacterial community composition of the bull kelp microbiome differs across coastal Washington [26], where the southernmost population in Puget Sound has decreased microbial diversity and abundance compared with a robust outer coast population at Tatoosh Island, quantified with both imaging [50] and metagenomics [51].

**Figure 1.**
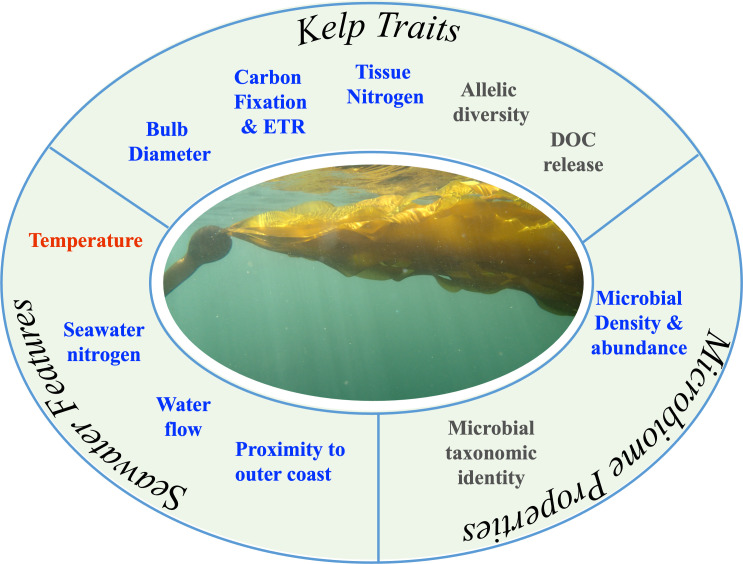
The fitness and persistence of *Nereocystis luetkeana* is linked to intrinsic kelp traits, seawater features and microbiome properties. Blue text represents factors in this study that are hypothesized to be linked positively to kelp fitness, while red denotes a negative correlation and grey text denotes either a variable or poorly understood relationship. ETR refers to electron transport rate. Photo by C. Pfister.

We quantified correlates of kelp traits, seawater features and properties of the microbiome that contributed to a geographic gradient of *Nereocystis luetkeana* health. Figure 1 shows the aspects of each that might contribute to *Nereocystis luetkeana* growth and persistence and the hypothesized relationship based on results reported here and in previous studies. Kelp traits that might contribute positively include carbon fixation, electron transport rate and tissue nitrogen. Although dissolved organic carbon (DOC) release could signal nitrogen-depleted tissue, its overall relationship to kelp health remains poorly understood. The diameter of the bulb, or pneumatocyst, may scale with blade mass and thus too is probably a correlate of reproductive output based on other studies of kelp size and reproductive investment [52,53]. Though untested here, genetic diversity is also expected to affect kelp fitness positively [54]. Seawater features that are hypothesized to contribute positively to kelp fitness are nutrient concentration, distance from the outer coast (a source of upwelling) and water flow, while increasing seawater temperature is a detriment. Features of the microbiome included microbial density, hypothesized to be a positive correlate, while the contributions of individual microbial taxa are still relatively poorly understood. Geographic differences in seawater features, kelp traits and microbiome properties allowed us to quantify correlated variables as well as generate hypotheses about kelp fitness in nature and its relationship to changing ocean conditions.

## 2. Methods

### 2.1. *Nereocystis* performance assays

**Figure 2.**
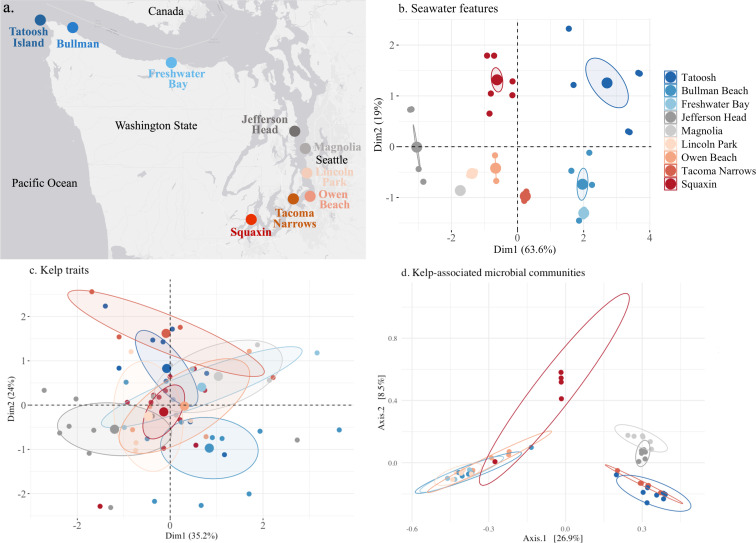
(a) The nine sites in Washington State, USA used in the study. In (b), multivariate representations of seawater features, (c) kelp traits and (d) kelp-associated microbial taxa, all represented with principal coordinate analysis (PCoA) of Bray–Curtis distance matrices with 95% confidence intervals. In (b), the seawater features included seawater temperature, salinity and the concentrations of dissolved inorganic nitrogen (DIN), DOC, PO_4_ and Si; sites differed based on permutational multivariate analysis of variance (PERMANOVA, *F*_8,102_ = 35.22, *p* < 0.001). In (c), the kelp traits included inorganic carbon fixation, dissolved organic carbon release, per cent nitrogen and carbon in tissues and the bulb diameter differed significantly (PERMANOVA, *F*_8,60_ = 6.91, *p* < 0.001). In (d), all microbial strains from all sites are represented (*F*_8,51_ = 8.45, *p* < 0.001). The data shown are not rarified, but rarefaction did not change the results.

We quantified carbon fixation, the release of DOC, and the tissue carbon and nitrogen content of bull kelp *Nereocystis luetkeana* (henceforth, *Nereocystis*) across nine sites ranging from the outer coast of Washington to the southernmost population in Puget Sound from 2019 to 2022 (figure 2a, electronic supplementary material, table S1). Included in our sampling is a kelp restoration site (Jefferson Head, also known as Doe Kag Wats) where *Nereocystis* has been grown from laboratory-cultivated individuals to repopulate a site where *Nereocystis* was locally extirpated. Kelp from eight sites were assayed in 2022, but the declining and perilous state of the Squaxin population did not allow us to take tissue and do these assays in 2022. We instead use data from Squaxin in 2019, 2020 and 2021. We also supplement data from other sites with data from 2019, 2020 and 2021.

At each site, a mature individual bull kelp was identified haphazardly, and the bulb (pneumatocyst) diameter was measured at the maximum width with calipers. Two fronds were removed from the mature kelp; each frond was placed in a separate plastic bag and brought to shore. There, one frond was assessed for carbon uptake, DOC release and nutrient uptake, and the second was sampled for carbon and nitrogen content and swabbed for microbial 16S rRNA amplicon sequencing.

We quantified carbon fixation using a section of the youngest, basal frond tissue extending 24 to 30 cm from the bulb. It was weighed (wet mass in g) with a Pesola scale and placed in a 1 l Nalgene container [55]. The incubation chamber had a removable lid that was filled to the top with indigenous seawater from the kelp bed at each site and sealed shut. Chamber incubations were conducted outdoors during July and August, using natural ambient light. Natural light levels were highly variable in these coastal locales; coastal fog, cloud cover and full sun occurred. We covered the chambers with a mesh screen to keep photosynthetically active radiation (PAR) in the range of 300−600 µM during the afternoon hours, thus mimicking the shallow depths where bull kelp fronds are found. Chambers were kept at 11−14°C, using a water bath cooled with ice packs or ice; each chamber was gently shaken every 10 min during a 1 h incubation to prevent boundary-layer conditions. With these methods, we were able to assay kelp within several hours of collection. Oxygen evolution inside the chamber was measured in real time using Pyro Science contactless fibre-optic sensors (Oxygen Sensor Spots OXSP5) and an optical oxygen and temperature meter (FireStingO2 FSO2-4), with the sensor spots affixed inside each 1 l chamber. Because the instrument has four channels, all replicates were run in groups of four with every set containing a seawater control to correct for oxygen changes due to plankton photosynthesis and respiration in freshly collected seawater. We chose not to filter seawater to maintain natural conditions but assessed the background activity of water column phototrophs with the seawater controls. Using a 1 : 1 molar ratio of O_2_-evolved : C-fixed based on the stoichiometry of photosynthesis, we estimated the amount of C-fixed per g dry mass of kelp per h for each individual at each site (*n* = 8−35).

The uptake or release of nutrients, including nitrate, nitrite, ammonium, phosphorus, silica and DOC during the 1 h incubation was estimated by nutrient measurement at the beginning and the end of the 1 h incubation in at least four replicates. Water was filtered through a GF/F filter and immediately frozen for analysis at the University of Washington Marine Chemistry Laboratory (methods from UNESCO [56]), with DOC samples frozen in 40 ml glass vials with Teflon caps (Shimadzu, VOA) and other nutrients in acid-washed 60 ml high density polyethylene (HDPE) Nalgene bottles.

The percentage of *Nereocystis* tissue mass composed of nitrogen and carbon was quantified for each individual blade. A 3 × 3 cm section of the meristem at approximately 20 cm from the base was excised, placed in foil, immediately chilled in the field, and then placed in a drying oven within 6 h of collection and dried to a constant mass for 48 h at 50°C. We quantified the per cent nitrogen and carbon in the meristematic region to standardize across individuals and populations and to minimize the effect of epiphytes and bacteria in this region of relatively new tissue. At Tatoosh Island, samples were initially dried in the rafters of a building, then transferred to a drying oven. Tissue was ground to a fine powder in a GenoGrinder Spex (Metuchen, NJ), weighed in aliquots of 1.2 to 1.5 mg and packed into 3.5 × 5 mm aluminium tins (Costech, Valencia, CA). Packed tins were analysed on an elemental analyser isotope ratio mass spectrometer at Northwestern University Stable Isotope Biogeochemistry Laboratory (NUSIBL).

### 2.2. The photosystem characteristics of the bull kelp

We tested whether individuals in populations at either end of the geographic distribution and environmental gradient, Tatoosh Island versus Squaxin Island, differed in their photosystem characteristics. Using pulse amplitude modulated (PAM) fluorometry *in situ* (Diving Pam, Walz, Germany) we estimated fluorescence over a range of irradiances. PAM is a non-invasive, *in situ* method that quantifies Photosystem II fluorescence parameters across different light levels. We followed the sampling procedure described in [55,57]; tissue was dark-adapted for 20 min and then rapid light curves were estimated with nine PAR levels from 0 to 1200 [58]. We estimated the maximum electron transport rate (ETR_max_) and the photosynthetic efficiency at low light, both as the initial linear slope of ETR to irradiance (*α*) and as the quantum yield of photosystem II (Yield) on nine individual *Nereocystis* at both Tatoosh Island and Squaxin Island, the populations that span the greatest range of environmental conditions. Tatoosh Island was assayed on 9 and 20 June 2019 and Squaxin Island on 2 July 2019. At both sites, we selected individuals at least 1 m apart at low tide.

### 2.3. The kelp bed environment

We assessed the temperature, salinity and nutrient concentrations of seawater at each site at the time of kelp and microbial collections. The salinity and seawater temperatures were assessed with a Castaway-CTD (Sontek) from a boat or kayak at all sites except Tatoosh Island where instrumentation is moored (Hach DS5, [40,59]). The seawater nutrients (nitrate, nitrite, ammonium, phosphorus, silica and DOC) were collected using the methodology described above for the incubations, sampling the water collected for the experiments immediately prior to the start of the experiments with at least two samples at each sampling date. Water nutrient sampling followed methods in Wootton *et al*. [59] and Pfister *et al*. [38], where surface seawater was collected in a 60 ml syringe, forced through a 25 mm GF/F filter and frozen for analysis at the University of Washington Marine Chemistry Laboratory.

### 2.4. The bacterial community on *Nereocystis* blades

We hypothesized that differences in *Nereocystis* host condition and surrounding seawater would change the microbial community composition residing on *Nereocystis* blades. The second blade collected from each individual kelp was sampled for microbes by rubbing a cotton swab for 20−30 s over the mid-blade region. The swab was placed in a 2 ml Eppendorf tube, immediately chilled and transferred to a freezer within 4−6 h. DNA from the swabs was extracted with a Qiagen DNeasy PowerSoil Kit (Qiagen). DNA was amplified, sequenced and amplicon sequence variants (ASVs) were identified by the Duchossois Family Institute Microbial Metagenomics Facility (DFIMMF) at The University of Chicago. The V4–V5 region within the 16S ribosomal RNA (rRNA) gene was amplified using universal bacterial primers and polymerase chain reaction (PCR) conditions described in [33]. Approximately 412 bp region amplicons were then purified using a spin column-based method (Minelute, Qiagen), quantified and dual index adapters were ligated. Sequences were generated from the Illumina MiSeq platform using the QIASeq 1-step amplicon kit (Qiagen) for generating libraries and using 2 × 250 paired end reads with 5000 to 10 000 reads per sample.

The default pipeline for processing MiSeq 16S rRNA reads was dada2 (v. 1.18.0) with minor modifications in R (v. 4.0.3). Reads were first trimmed at 190 bp for both forward and reverse reads to remove low-quality nucleotides, and chimaeras were detected and removed using the default consensus method in the dada2 pipeline. Then, ASVs with length between 320 and 365 bp were retained. Taxonomy of the resultant ASVs were assigned to the genus level using the RDP classifier (v. 2.13) with a minimum bootstrap confidence score of 80. Species-level classification used blastn (v. 2.13.0) and the refseq_rna database.

We analysed ASVs with R, phyloseq and microViz (R version 2023.06.2+561). Chloroplast DNA that was not classified as Cyanobacteria were generally diatoms (Bacillarophyta) and were filtered out, as well as any other chloroplast sequence that did not match to the class Cyanobacteria. Sequences of corn (Zea, in Plantae), in low numbers at some Puget Sound sites, were also removed.

We pruned ASVs that occurred only twice across all samples, considering that with *n* = 3–8 at each site, we should expect to see a sequence at least three times. The quantity of DNA varied across samples and sites. Some samples from Puget Sound had low DNA concentrations, consistent with imaging at some sites that showed low diversity and abundance of microbes on Squaxin Island samples [50]. We thus did not rarify data to examine alpha and beta diversity, so as not to compromise the information we had from sites where microbial abundance might be low.

We tested what taxa drove geographic distinctness in microbial abundance with SIMPER analyses, a means of testing what microbial taxa in each site contributed the most to beta diversity metrics [60]. We visualized with a heat map,using rarefied data to minimize sequencing depth effects on these analyses. Each taxa’s contribution is relative and the sum of all taxa that contributed differences summed to 1 for each set of paired comparisons. We quantified the taxa that contributed the most to geographic distinctness by focusing on only those taxa that contributed the first 50% (cumulative probability of 0.5) of the difference and were significant at a stringent *p* < 0.001.

We further tested correlates of individual kelp traits with these top 10 abundant and distinct genera, testing whether individual carbon fixation rates, the per cent tissue nitrogen or the diameter of the *Nereocystis* bulb was related to the number of sequences that were classified in each genus, summing over different strains within a genus.

We visualized site differences in (i) features of seawater, (ii) kelp traits, and (iii) microbial communities on kelp blades with principal coordinate analysis (PCoA) and quantified the differences based on the Bray–Curtis distance metric. In most cases, kelp traits were measured on the same individual kelp where we sequenced and quantified the blade microbes, providing the opportunity to link host traits with microbial communities. Where there was not an identical correspondence between kelp individuals and microbial sampling, due to occasional PCR or sequencing issues, we used swabs from kelp individuals measured in the same bed on the same date. There were six seawater features measured: surface seawater temperature, salinity, the concentration of dissolved inorganic nitrogen (DIN), DOC, phosphate and silica as silicic acid. Kelp traits included the inorganic carbon fixed and the DOC released per unit kelp mass per hour, the percentage of nitrogen and carbon in kelp tissues, and the bulb diameter. The matrix of microbial composition contained the ASVs to the strain level.

The resulting three matrices of data included kelp traits, seawater features and microbial composition. The effect of site was tested with permutational multivariate analysis of variance (PERMANOVA; adonis2, *vegan* in R). We further used these three distance matrices to test whether kelp traits correlated with seawater features or microbial composition by quantifying the linear correlation between matrices of data with a Mantel test (ade4, 999 permutations, [61]).

Finally, we tested whether the microbial density on individual kelp blades at either end of the geographic distribution, Tatoosh Island versus Squaxin Island, differed using 4,6-diamidino-2-phenylindole (DAPI) fluorescent staining of bacterial cells. We quantified bacterial cells from a section of the blade at approximately 20 cm distal from where the blade articulates with the bulb. We sampled a 3−5 mm slice from 10 individuals at each site (Tatoosh on 4 August 2019, Squaxin on 2 July 2019), preserving each slice immediately in Formalin, transferring to 50 : 50 EtOH : phosphate-buffered saline (PBS) after 1 h, then freezing [62]. In the laboratory, each piece was again sliced with a razor blade to approximately 0.5 mm, placed on a glass slide, and a solution of 1 µg DAPI : 1 ml PBS was dropped on the slide-mounted slice. The slide was kept in dark for 20 min, chilled and rinsed with PBS prior to visualizing at 100× with an Olympus BX50 fluorescence microscope with a DAPI filter. We photographed 10 fields of view, selected haphazardly, for each individual slice, resulting in 100 images at each Tatoosh and Squaxin. In samples where bacteria were sparse or non-existent, we repeatedly adjusted the fine focus to verify no bacteria were present. We quantified the bacteria in ImageJ, enumerating the cells in the blue channel, adjusting the background and thresholds identically for all 200 images to eliminate autofluorescence by kelp cells. We calculated bacterial cell density as a percentage of the total imaged area.

## 3. Results

### 3.1. Environmental, seawater features

The seawater features of temperature, salinity and nutrients (DIN, DOC, PO_4_, Si) displayed strong distinctions among sites (figure 2b). The concentration of DIN was greatest at the westernmost sites of Tatoosh Island and Bullman Beach and lower in Puget Sound (electronic supplementary material, figure S12). Puget Sound sites had a lower overall mean DIN concentration (*F*_1,74_ = 25.93, *p* < 0.001). PO_4_ and Si showed similar patterns and differed among sites, especially Tatoosh Island and Jefferson Head; increased concentrations at south Puget Sound sites were suggested with only marginal values of significance between regions (*p* = 0.062 and 0.069, respectively for PO_4_ and Si, electronic supplementary material, table S3). Temperature tended to increase while salinity and DIN decreased from west to southernmost sites in Puget Sound.

### 3.2. *Nereocystis luetkeana* traits

**Figure 3.**
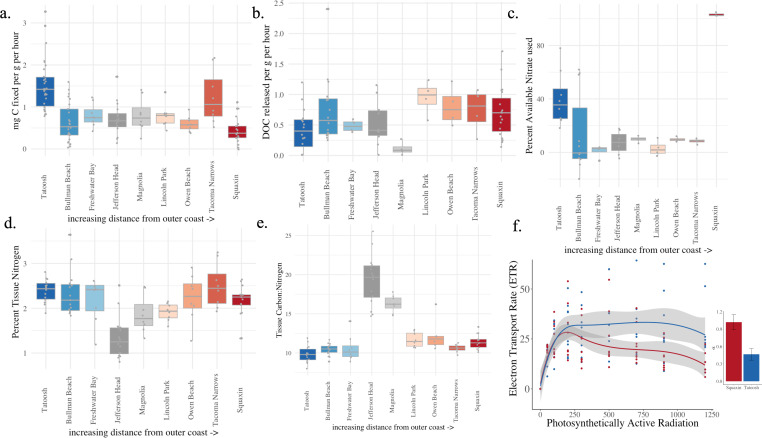
The traits of individual kelp across the nine sites include (a) carbon fixed and (b) dissolved organic carbon (DOC) released (mgC per g dry mass of *Nereocystis* blade tissue per h), (c) nitrate uptake within 1 h as a percentage, (d) the per cent tissue nitrogen by dry mass, (e) the carbon : nitrogen ratio in blade tissues, and (f) the electron transport rate (ETR), a measure of photosynthetic activity, as a function of light from both Tatoosh and Squaxin. The inset shows that Squaxin *Nereocystis* had a higher *α*, or initial linear slope increase in photosynthetic activity at low light (*F*_1,16_ = 11.37, *p* = 0.004). Boxplots show the interquartile range and the median; all points are shown. The sites correspond to figure 2a. The statistics are shown in electronic supplementary material, table S3.

Carbon fixation varied across the range of *Nereocystis* and mean C fixation (mg C per g dry mass per h) was more than three times greater at the outer coast site of Tatoosh (1.50) compared with the lowest estimate at Squaxin, the most southern site in Puget Sound (0.48) (electronic supplementary material, table S2). Tatoosh Island and Tacoma Narrows had the greatest rates and were statistically indistinguishable (figure 3a, ANOVA and Tukey honestly significant difference (HSD), *F*_8,126_ = 12.99, *p* < 0.001, electronic supplementary material, table S3). Despite the relatively high rates at Tacoma Narrows, there were regional differences; the six populations in the Puget Sound region had a lower mean carbon fixation rate (0.69 across all six populations) compared with those along the Strait of Juan de Fuca (1.09, *F*_1,133_ = 17.29, *p* < 0.001, electronic supplementary material, table S3).

The mean release of DOC was greatest at Lincoln Park (0.95 mg DOC per g dry mass per h) and least at Magnolia (0.11 mg) (electronic supplementary material, table S2). Differences among sites in DOC release were indicated (figure 3b, ANOVA, *F*_8,71_ = 2.64, *p* = 0.014, electronic supplementary material, table S3), though post hoc tests did not indicate strong distinctions among the sites (Tukey’s HSD). The mean of the six Puget Sound populations did not differ in their DOC release rate (0.65) compared with those in the Strait of Juan de Fuca (0.57, *F*_1,78_ = 1.77, *p* = 0.187). Though DOC release alone did not differ greatly across sites, the ratio of carbon fixed to dissolved carbon released differed among sites (*F*_8,72_ = 2.53, *p* = 0.018) and regions (*F*_1,79_ = 4.27, *p* = 0.042). The C fixed : DOC release ratio for bull kelp at Tatoosh was an order of magnitude higher than other sites, largely because some DOC release rates there were near zero. Nitrate uptake rates over the 1 h incubation revealed that the two populations separated by the greatest distance (Tatoosh and Squaxin) had the highest nitrate use as a percentage of the nitrate available (figure 3c, electronic supplementary material, table S3).

The nitrogen content of bull kelp blades differed among sites in Puget Sound; the populations of Magnolia, Lincoln Park and Jefferson Head had the lowest nitrogen content while all the other populations were greater and statistically indistinguishable (figure 3d, *F*_8,143_ = 11.35, *p* < 0.001 and Tukey’s HSD). Tacoma Narrows and Tatoosh Island kelp had 2.50% and 2.39% tissue nitrogen, respectively, while Magnolia, Lincoln Park and Jefferson Head were all under 2.00% (electronic supplementary material, table S1). Regional differences were indicated with a mean tissue nitrogen of 1.93% for Puget Sound populations compared with the 2.29% of three populations along the Strait of Juan de Fuca (electronic supplementary material, table S3). Correspondingly, the C : N ratio of tissue was highest at Jefferson and Magnolia (figure 3e) and populations within Puget Sound had an increased C : N (13.92) compared with other locales (10.24), a key regional difference between Puget Sound and outer coast populations that was statistically significant (*F*_1,94_ = 32.5, *p* < 0.001).

When carbon fixation, bulb size, per cent nitrogen and per cent carbon (electronic supplementary material, table S3) were combined in a multivariate trait analysis, there were significant site differences (figure 2c, PERMANOVA, *p* < 0.001 with adonis2 in R ‘vegan’).

### 3.3. Photosynthetic features

The most geographically distant populations, Tatoosh Island and Squaxin Island also differed in photosystem traits estimated with PAM fluorometry (figure 3f). Kelp blades from the southernmost site in Puget Sound (Squaxin) demonstrated an increased *α*, or initial linear slope with irradiance (figure 3f, *F*_1,16_ = 11.37, *p* = 0.004), and a yield (*F*_*v*_/*F*_*m*_) that showed a trend to be greater than at Tatoosh Island (*F*_1,16_ = 4.37, *p* = 0.053). Squaxin kelp also showed a lower onset of light saturation (*E*_*k*_, a mean of 28.1 versus 93.4, *F*_1,16_ = 8.44, *p* = 0.010), while maximum ETRs were indistinguishable at low light levels (*F*_1,16_ = 1.79, *p* = 0.201) even though *Nereocystis* at Tatoosh showed increased electron transport at higher light levels.

### 3.4. Microbial associates of *Nereocystis luetkeana*

Microbial community composition on the blades of *Nereocystis* showed geographic distinctness in ASV diversity (figure 2d), but also differences at the phylum, class and genus level (electronic supplementary material, figure S2). The number of reads varied across samples from 1509 to 82 227 with a mean of 19 512. We found 623 ASVs across all non-rarified samples in 14 phyla. The four phyla of Bacteroidetes, Planctomycetes, Proteobacteria and Verrucomicrobia made up 91% of the ASVs shared across all sites (electronic supplementary material, figure S2a and table S4). Although class and genus level composition could vary greatly across sites (electronic supplementary material, figure S2b,c), 11 genera were present in 80% of all the samples collected and included *Luteolibacter, Rubritalea, Flavilitoribacter, Hellea, Dokdonia, Granulosicoccus, Marinoblastus, Persicirhabdus, Litorimonas, Aquimarina* and *Thalassotalea*, and all were in the most abundant ranks (electronic supplementary material, figure S2c). These 11 genera that dominated at each site included at least one representative from one of the four most abundant phyla.

Microbial beta-diversity patterns show strong patterns across sites, with the central Puget Sound sites of Magnolia and Jefferson Head clustering together, and the Puget Sound sites of Lincoln Park and Owen Beach clustering with the Strait of Juan de Fuca sites of Bullman Beach and Freshwater Bay (figure 2d), while Squaxin Island maintained a relatively distinct bacterial community. Two geographically separate, but environmentally similar sites, Tatoosh Island and Tacoma Narrows, had strong microbial community similarity. The community differences shown in figure 2d were unchanged when samples were rarified to 1159 ASVs.

**Figure 4.**
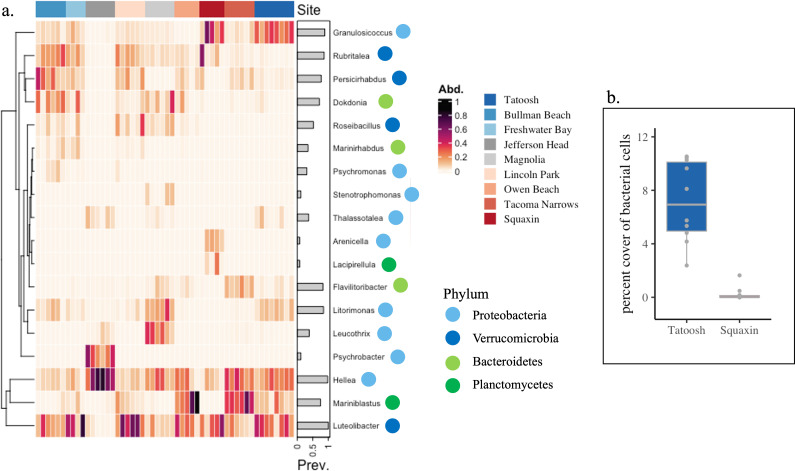
(a) The 50 ASVs with the greatest relative abundance grouped by genus and by site [63], and (b) the abundance of bacterial cells expressed as a per cent cover and estimated with DAPI staining at Tatoosh and Squaxin Islands. Tatoosh Island had higher bacterial density (7.17 versus 0.23%, *F*_1,18_ = 53.2, *p* < 0.001, *n* = 10 at each site).

An analysis of the ASVs showed that few taxa contributed disproportionately to the differences among sites (SIMPER, figure 4, electronic supplementary material, figure S5); only 1−7 taxa determined most of the taxonomic distinction and these taxa were within the 10 most abundant genera across all sites. Planctomycete genera *Mariniblastus* and *Lacipirellula* were unique to Tacoma Narrows, Owen Beach and Squaxin, while the Gammaproteobacteria *Psychrobacter* distinguished the restoration kelp bed at Jefferson Head, and *Granulosiccocus* was significant at Tatoosh, Freshwater Bay and Squaxin. The Flavobacteriia *Dokdonia* (phylum Bacteroidota) was represented disproportionately at the two sites in the Strait of Juan de Fuca (Bullman Beach and Freshwater Bay) and Magnolia. Although Verrucomicrobia were generally widespread, several key taxa within this phylum were notably absent at the Puget Sound site of Jefferson Head (figure 4, electronic supplementary material, figure S2). *Flavilitoribacter* (Bacteroidetes) was relatively unique at Owen Beach and Tacoma Narrows.

The density of bacterial cells on *Nereocystis* blades enumerated with DAPI staining was almost an order of magnitude greater at Tatoosh Island (7.17% cover) compared with Squaxin (0.23%) (*F*_1,18_ = 52.3, *p* < 0.001, *n* = 10 each, figure 3b). Across the 100 images we took at each site, only 12 had no bacterial cells visible at Tatoosh, while 71 photos had no bacterial cells visible at Squaxin. Electronic supplementary material, figure S3 shows representative microscope images from each site, including fields of view with no visible bacteria (electronic supplementary material, figure S3e,f).

### 3.5. Relating seawater features, kelp traits and microbial components

There was a significant linear correlation between distance matrices of seawater features with microbial ASVs (Mantel test, *r* = 0.133, *p* = 0.029), indicating that similarities and differences estimated for seawater features were reflected in microbial community composition (a comparison of figure 2b with 2d). For example, the seawater features at Squaxin, Jefferson Head and Magnolia are distinct, as are the microbial communities at these sites. By contrast, the matrix of seawater features and kelp traits (figure 2b versus 2c) were uncorrelated with a Mantel test (*r* = −0.005, *p* = 0.569) and kelp traits and microbes (figure 2*c* versus 2d) were also uncorrelated (*r* = −0.085, *p* = 0.872).

Although strong relationships of kelp traits with seawater features or kelp traits with microbial community structure were not indicated in the multivariate data and analysis, kelp traits and seawater features did show relationships when environmental variables were tested in isolation. Carbon fixation, per cent nitrogen and bulb diameter were positively correlated with the concentration of DIN and phosphate (PO_4_), and negatively correlated with seawater temperature (electronic supplementary material, figure S4 and table S5). In general, geography was a strong determinant, with lower kelp condition metrics and nutrient concentrations in central Puget Sound, and enhanced kelp condition and nutrient concentrations in the western Strait of Juan de Fuca.

Kelp traits, including carbon fixation rates, the per cent tissue nitrogen, and the diameter of the *Nereocystis* bulb (electronic supplementary material, figure S6 and table S6), also correlated with some abundant bacterial taxa and seawater features. Taxa that correlated positively with individual kelp fitness proxies of carbon fixation and per cent nitrogen included *Hellea* in the class Alphaproteobacteria, *Mariniblastus* in the phylum Planctomycetes, *Flavilitoribacter* in the class Saprospira, and the phylum Verrucomicrobia representatives *Rubritalea* and *Persicirhabdus*. The Gammaproteobacteria *Psychrobacter* correlated negatively with per cent nitrogen, largely due to its abundance on the nitrogen-poor Jefferson Head kelp.

## 4. Discussion

### 4.1. Kelp fitness across a geographic gradient

As outlined in figure 1, kelp traits related to fitness, including carbon fixation and tissue nitrogen showed strong patterns across Washington coastal waters and south into Puget Sound. Individuals from populations in central and south Puget Sound showed the lowest proxies for individual fitness-related traits (figure 3), and this low performance was correlated with higher seawater temperatures and lower DIN. Rates of carbon fixation and tissue nitrogen concentration were positively related to DIN (electronic supplementary material, figure S4), a result demonstrated previously at the geographically most distant two sites of Tatoosh Island and Squaxin Island [37]. Detrimental effects of increased seawater temperature on kelp photosynthesis and carbon fixation were suggested here and have been demonstrated experimentally [16]. Along north–south-oriented coastlines, latitude is a correlate of seawater temperatures and covaried positively with kelp size in the northeast Atlantic for the kelp *Laminaria hyperborea* [22]. Similarly, kelp in the Southern Hemisphere too have decreased photosynthesis at lower latitudes associated with increased temperature [64].

*Nereocystis* at Tacoma Narrows notably did not follow the geographic gradient of a decrease in fitness-related traits proceeding south into Puget Sound. Kelp productivity and nitrogen content was enhanced at Tacoma Narrows and showed marked similarity of kelp traits and microbial composition to Tatoosh Island. At Tacoma Narrows, there is a sill that separates the watershed [65], generating strong currents and localized upwelling of cold, nutrient-rich seawater [42]. The smaller bulb size recorded at Tacoma Narrows may reflect an adaptive response to these high currents, streamlining the kelp [66]. Tacoma Narrows has lower temperatures and high DIN concentrations that reflect the sill-generated water movement compared with sites further south into Puget Sound [47]. Thus, some geographic patterns in kelp health are probably generated by physical features that bring cold seawater to the surface. Though overall DIN was low at Squaxin Island (electronic supplementary material, table S2), there was relatively increased ammonium at this site (electronic supplementary material, figure S1) that may maintain kelp growth.

Kelps may interact with nutrients in the environment through several routes. Sites with low concentrations of DIN and phosphorus may restrict kelp photosynthesis and growth, both over the short interval that we assessed carbon fixation (figure 3) and throughout the growing season. Even though kelp might use alternative nitrogen sources when nitrogen is limiting, including dissolved organic nitrogen (DON) [32,67] may not compensate for low DIN, and kelp may be dependent upon associated microbial nitrogen transformation when DIN is scarce [32,68]. Low concentrations of DIN may be associated with a reduced ability to withstand high temperature stress in kelp, as demonstrated in the giant kelp *Macrocystis pyrifera* [69,70]. However, manipulative experiments with the species studied here, *Nereocystis luetkeana,* demonstrated that enhanced nutrients cannot ameliorate stress from elevated temperature [16,71]. Our findings here also suggest that kelp fitness positively correlates with lower temperatures and higher DIN concentration, even though we could not separate the contributions of each variable to kelp fitness (figure 1). Covarying with temperature and DIN are patterns of genetic variation. Central and southern Puget Sound bull kelp are characterized by decreased allelic variation compared with bull kelp populations in the Strait of Juan de Fuca [72,73], raising the possibility that host fitness is related to the degree of inbreeding or genetic drift [73]. The extent to which local-scale seawater temperatures and nutrient levels, in combination with genetic background, determine kelp fitness traits is an important area of future research as genomic data is combined with local-scale environmental data.

Whether the kelp *Nereocystis luetkeana* is acclimatized or adapted to local conditions is unknown, though the photosynthetic physiology of kelp at the southern limit to their range in Puget Sound (Squaxin) suggests acclimatization to lower light levels [74], or possibly adaptation. Compared with *Nereocystis* at Tatoosh Island, *Nereocystis* individuals at Squaxin had higher photosystem yield and a greater initial slope at low irradiance, though had light saturation and decreased electron transport as light level increased. Thus, bull kelp at Tatoosh Island have greater photosynthetic activity at higher light, a trait that may contribute to the increased carbon fixation by *Nereocystis* at Tatoosh Island. *Nereocystis* at Squaxin and other Puget Sound sites often remain submerged except at low tides, whereas populations within the Strait of Juan de Fuca and at Tatoosh Island have biomass at the water surface at most tide levels (personal observation). Whether this is photoprotective, a response to strong currents, or simply the result of slower growth rates is unknown, though the net result is that kelp in Puget Sound that are often submerged have decreased access to PAR. *In situ* environmental monitoring is needed in the region to understand the extent to which differences in water column particulates and turbidity drive these patterns.

### 4.2. Insights from individual kelp traits

Globally, there is increased capacity to assess kelp populations with remote sensing methodologies [75]. Remote sensing includes drones, piloted aircraft and satellites, and allows large areas to be censused to make strong inference about population trends [46], with ongoing efforts to relate remotely derived data to local patterns of chlorophyll and carbon content [76]. Our local-scale assessment of kelp traits, including their microbiome, at the level of the individual provides insight into stressors. For example, despite the persistence of populations such as Lincoln Park and Magnolia, they were characterized by relatively low rates of carbon fixation, and an elevated ratio of dissolved organic carbon relative to the carbon they acquire through photosynthesis compared with *Nereocystis* in the Strait of Juan de Fuca (electronic supplementary material, table S3). While there is uncertainty in the relationship between kelp health and DOC release (figure 1), it may be a negative correlate of kelp fitness for several reasons. First, elevated levels of DOC release can be the result of ‘stoichiometric overflow’ [77] where kelp may not have enough nitrogen to maintain the carbon that is fixed [37], a phenomenon also hypothesized for root exudate in terrestrial plants [78] Second, kelp may also release DOC to prevent damage to the RUBISCO enzyme during nitrogen limitation [79]. The relatively high tissue C : N ratio at Jefferson Head and Magnolia provides further support of nitrogen limitation to photosynthesis [79], as does the relatively low DIN concentrations in these areas that have reduced kelp tissue nitrogen. Thus, the persistence of populations such as these in aerial (remote) surveys may belie their vulnerability to further environmental change. Similarly, the Squaxin population is in steep decline [47], despite its comparatively high tissue nitrogen concentration and its ability to take up nitrate in experimental chambers. Yet, stress in Squaxin kelp may be indicated by the lower carbon fixation rates compared with individuals at other sites, and suggests it may be inhibited from using the tissue nitrogen due to elevated temperatures [71] or reduced access to sunlight from turbidity [19,80] or competing algae [81]. Indeed, the reduced photosystem capabilities indicated at Squaxin compared with Tatoosh Island suggests adaptation or acclimatization to unique conditions, while potentially near its physiological limits. An increased understanding of how patterns in remote sensing data do or do not indicate future trajectories of kelp beds with changing local conditions is needed.

### 4.3. Microbial response to host and environmental features

In general, our analyses of the microbial community showed many taxa common to kelp in Washington coastal waters [26,31,33] and taxa demonstrated to be present globally [82–85], including the genus *Granulosicoccus* [28,86,87]. Our demonstration that 11 genera were present in at least 80% of all swabbed kelp surfaces suggests they are members of a core group of microbial taxa and is consistent with a growing number of studies that show that seaweeds have core taxa [88,89].

Microbial communities, kelp traits and features of seawater changed along the geographic gradient and differed by site (figure 2). Although seawater features were not strongly related to kelp traits based on a multivariate Mantel test, univariate tests did reveal correlations, with DIN, PO_4_ and seawater temperature correlating with bulb diameter, carbon fixation and tissue nitrogen (electronic supplementary material, figure S4) and illustrated in figure 1. In turn, these kelp traits were correlated with some bacterial taxa (electronic supplementary material, figure S6). Nutrients are a persistent determinant of kelp fitness globally [90], and seawater nutrient content often covaries negatively with seawater temperature, especially along upwelling shores [40]. Experimental manipulation of temperature and nutrients with *Nereocystis* showed that temperature had a greater effect than nutrients on bull kelp growth and survival [16] and on disrupting microbial community structure [34].

The strong covariation between kelp traits and geographic locale that we observed makes it difficult to solely test the role of host condition on microbial assemblages [8] and highlights the complex interactions between seawater features, kelp traits and microbial interactions that we portray as single factors in figure 1. Despite strong spatial patterns in kelp and microbes, there was similarity between Tatoosh Island and Tacoma Narrows kelp traits (figure 1b) and microbial composition (figure 1d), even though the two sites are separated by more than 200 km of shoreline. The persistent upwelling of seawater at Tacoma Narrows, generated by a sill [65], may provide a seawater environment at Tacoma Narrows similar to that at Tatoosh Island, where the mouth of the Strait of Juan de Fuca is also an area of intense mixing and upwelling [91,92], perhaps resulting in the delivery of similar microbial taxa that are associated with upwelling water. The similarity of the *Nereocystis* microbiome at Tatoosh and Tacoma Narrows, despite distinction in host genotypes [73], and the similarity of the microbial community sites in Central Puget Sound (Jefferson Head, Magnolia, Lincoln Park) suggest that the environment may select for similar microbial communities, a result also suggested for two other species of kelp in the region [83]. While kelp-associated microbial communities appear to be influenced by features of the surrounding water column, our analyses of the dominant taxa and community composition across sites revealed that host attributes also influence the bacterial taxa that colonized the kelp host.

Bacterial density differed between the two sites separated by the greatest distance, with Tatoosh having the highest density (figure 4b), echoing results of [50] that showed sparse bacterial cells from CLASI-FISH imaging of *Nereocystis* from Squaxin. Although we only quantified bacterial cell density on a single date, our results are also consistent with our DNA extraction results in this study and previous studies, where DNA extracts were typically lower at Squaxin, a population in decline, than Tatoosh Island [51], where carbon fixation and tissue nitrogen were greatest (figure 2). Despite the repeated suggestion that bacterial cell density is lower at Squaxin compared with Tatoosh, the microbial metabolism of ammonification was greater at Squaxin [32] where ammonium concentrations were relatively elevated (electronic supplementary material, figure S1). While marine hosts such as sponges and fish have been shown to vary in the density of bacteria they host, this has been shown at the species level, not the population level [93,94]. The connection between microbial cell density, the function and metabolisms of individual microbial taxa, and host fitness is poorly understood and deserves increased attention.

Although there was not strong correspondence of kelp traits and the matrix of microbial ASV composition, bacterial ASVs differed across geographically separated sites. Individual bacterial taxa correlated with traits measured on individual kelp, including *Psychrobacter*, a Gammaproteobacteria, that was abundant on the restoration kelp at Jefferson Head, a site with relatively low carbon fixation. Though the driver of this correlation is unknown, alginate lyases are present in *Psychrobacter* and it may benefit from the relatively high DOC release rates and high carbon content of Jefferson Head kelp [95].

The Planctomycetes that were increased in relative abundance in Puget Sound sites may have importance for their hosts through their use of sulfatases to metabolize sulfated polysaccharides that are released from seaweeds [96]. Verrucomicrobia too are thought to be important degraders of complex kelp polysaccharides, including fucoidan [97]. Members of the Verrucomicrobia, *Rubritalea* and *Persicirhabdus*, showed positive correlations to tissue nitrogen content and bulb diameter, suggesting that kelp exudates may provision these microbes. *Mariniblastus* displayed relatively higher abundance at Tacoma Narrows and Owen Beach, and has been associated with increased water temperature [98].

Overall, *Nereocystis* showed strong geographic patterns in host traits, with increased carbon fixation and nutrient content in areas along the Strait of Juan de Fuca and lower values into Puget Sound that followed an environmental gradient of increasing temperature and decreasing nutrients. Bacterial composition also differed among sites, and some bacterial taxa were related to both kelp traits and seawater features. The multivariate nature of seawater features, host traits and microbial community composition makes correlative relationships difficult to detect, necessitating future studies that link specific environmental features with microbial taxa and host.

## Data Availability

All data are uploaded to Dryad [99]. All raw DNA sequence data are available at NCBI, PRJNA1301461. Supplementary material is available online [100].
